# SARS-CoV-2 vaccine alleviates disease burden and severity in liver transplant recipients even with low antibody titers

**DOI:** 10.1097/HC9.0000000000000025

**Published:** 2023-02-01

**Authors:** Abed Khalaileh, Ashraf Imam, Alaa Jammal, David Hakimian, Johnny Amer, Asher Shafrir, Yael Milgrom, Muhammad Massarwa, Wadi Hazou, Majd Khader, Rifaat Safadi

**Affiliations:** 1 Faculty of Medicine, Hebrew University of Jerusalem, Jerusalem, Israel; 2 Department of Surgery, Hadassah Medical Center, Jerusalem, Israel; 3 The Liver Institute, Hadassah Medical Center, Jerusalem, Israel

## Abstract

We retrospectively assessed the clinical Pfizer’s mRNA SARS-CoV-2 BNT162b2 vaccination outcomes and the serologic impact on liver transplant (LT) recipients.

**Patients and Methods:**

One hundred and sixty-seven LT cases followed between March 1, 2020 and September 25, 2021, and were stratified into two groups: (1) 37 LT recipients after SARS-CoV-2 infection before vaccine era and (2) 130 LT recipients vaccinated with 2 doses without earlier SARS-CoV-2 exposure. Serum SARS-CoV-2 spike immunoglobulins (anti-S) were assessed 7 days following vaccination (Liaison assay).

**Results:**

In addition to the 37 nonvaccinated cases (22.2% of total group) who experienced SARS-CoV-2 infection (34 symptomatic and 3 asymptomatic), another 8 vaccinated symptomatic recipients (4.8%) were infected (5 from the third and three from the fourth waves). Three of the 45 infected cases died (6.7%) before the vaccine program. Vaccinated group: of the 130 LT vaccinated recipients, 8 (6.2%) got infected postvaccination (added to the infected group) and were defined as clinical vaccine failure; 38 (29.2%) were serological vaccine failure (total failure 35.4%), and 64.6% cases were serological vaccine responders (anti-S≥19 AU/mL). Longer post-LT interval and lower consumption of immunosuppressants (steroids, FK506, and mycophenolate mofetil) correlated with favorable SARS-CoV-2 vaccine response. Mammalian target of rapamycin inhibitors improved vaccine outcomes associated with lower FK506 dosages and serum levels. Patients with anti-S levels <100 AU/mL risked losing serologic response or being infected with SARS-CoV-2. A booster dose achieved an effective serologic response in a third of failures and most responders, securing better and possibly longer protection.

**Conclusion:**

Pfizer’s BNT162b2 vaccine seems to lessen SARS-CoV-2 morbidity and mortality of LT recipients even with weak serological immunogenicity. Switching mycophenolate mofetil to mammalian target of rapamycin inhibitors might be effective before boosters in vaccine failure cases. A booster vaccine should be considered for nonresponders and low-responders after the second dose.

## INTRODUCTION

Since the early stage of the SARS-CoV-2 pandemic,^1^ health organizations have emphasized the urgent need for an efficient vaccination program.^1^ The race for different vaccines began promptly.^2^ Several have now been approved, and a few more are in the companies’ pipelines, in the late clinical trial stage.^3^

Israel initiated a vaccination program on December 19, 2020, for all ages ≥16 years and later for SARS-CoV-2 naive subjects >12 years old. More than 93% of the eligible population received at least 1 dose, and 89% completed both doses of the Pfizer vaccine.^4^ The Israeli vaccination experience suggests that the Pfizer’s BNT162b2 vaccine is effective for a wide range of SARS-CoV-2-related outcomes, consistent with the randomized trial.^5^

On July 12, 2021, the administration of a third (booster) dose of the Pfizer-BioNTech vaccine was approved in Israel for immunosuppressed individuals, particularly solid organ recipients (SOTR). On July 30, 2021, the vaccine was approved for all persons 60 years or older who received a second vaccine dose at least 5 months earlier.^6^ However, the age limit for the booster rapidly declined. Accordingly, we advised our recipients to follow the same national vaccination program, including the booster policy.

SARS-CoV-2 vaccines are safe in SOTR with a similar rate of adverse events as in the general population. Pfizer-BioNTech and Moderna SARS-CoV-2 vaccines were safe in 187 SOTR.^7^ However, SARS-CoV-2 vaccinations were less efficient in SOTR patients than in immunocompetent persons. Furthermore, the antibody response to 2 mRNA vaccine doses was lower. SOTR evaluation showed^8^ that 98/658 (15%) had a measurable antibody response after doses 1 and 2, 301 (46%) had no antibody response after both doses (vaccine failure), and 259 (39%) had no antibody response after dose 1, and a response after dose 2. This seroconversion rate in SOTR varied in different centers and organs.^9^ Factors for poor vaccine immunogenicity include older age, shorter time from transplantation, use of mycophenolate and belatacept, and worse allograft function. Liver transplant (LT) recipients also developed a weaker immune response to the 2 doses of the Pfizer’s BNT162b2 vaccine. Factors influencing serological responses include age, renal function, and immunosuppressive medications.^10^

In the current study, we show for the first time the clinical and serologic impact of 3 doses of the Pfizer-BioNTech vaccine in LT recipients. Despite the low antibody response, morbidity and mortality were alleviated in the vaccinated LT population.

## PATIENTS AND METHODS

### Local strategy according to the national policy

In the Liver Institute, Hadassah Medical Organization (HMO), all liver patients, are encouraged to receive SARS-CoV-2 vaccines as part of the national vaccination policy in Israel. Active LT recipients who have attended the liver clinic since early 2020 are routinely questioned about their history of SARS-CoV-2 infection or exposure and are asked to contact us if SARS-CoV-2 infection is suspected or validated.^11^

### Vaccine and vaccination dosages and serology

The Pfizer’s BNT162b2 vaccine consists of 2 doses (30 μg, 0.3 mL each) administered intramuscularly, 21 days apart.

Although vaccine efficacy is defined as protection from SARS-CoV-2 infection and not serologic response, it is commonly accepted that failure cases do not develop a serologic response. The measurement of serum antibody levels to assess vaccine efficacy is widely accepted in non-SARS-CoV-2 vaccines.^12^,^13^ The serologic SARS-CoV-2 spike immunoglobulins (anti-S) tests are widely used by the Israel.

The anti-S IgG was measured using the DiaSorin’s LIAISON kit^14^ at least 1 week after the second and third vaccination. Serum anti-S levels of ≥19 AU/mL were considered a good vaccine response, serum anti-S levels of <12 AU/mL were considered vaccine failure, and an equivocal response refers to serum anti-S levels of ≥12 and <19 AU/mL. The kit gave a range from no detectability to 400 AU/mL. Higher anti-S levels were recorded as >400 AU/mL.

### Immunosuppression protocol at HMO

Maximal immunosuppression is required early post-transplant when rejection risk is most significant. Maintenance immunosuppression regimens vary considerably. The most common regimens include (1) a calcineurin inhibitor (CNI, usually tacrolimus but also cyclosporine-A) with or without corticosteroids; (2) a CNI with an antimetabolite [azathioprine or mycophenolate mofetil (MMF)] with or without corticosteroids; (3) a CNI and mammalian target of rapamycin (mTOR) inhibitor (mTORi, usually everolimus but also sirolimus) with or without corticosteroids; (4) CNI-free regimens in rare cases based on mycophenolate or mTOR inhibitor or both (with or without corticosteroids).

The choice of the immunosuppressive regimen is dependent on many factors, including (1) time after transplant that starts as an induction regimen (<3 mo), to high-dose maintenance (<1 y) and can be reduced at later times after transplantation. The mTOR inhibitors (mTORi) are deferred for 3 months as they impair wound healing and increase the risk of hepatic artery thrombosis. (2) Indication for transplant, as long-term use of corticosteroids in autoimmune conditions needs lower tapering, whereas high-dose steroids promoted HCV replication. (3) Acute and/or chronic rejection episodes. (4) History or risk of cancer encouraging the mTORi regimens to reduce the risk of some de novo cancers. (5) Complications of previous immunosuppression. (6) Wish of recipient or partner to conceive and/or breastfeed (mycophenolate and mTORi are teratogenic). (7) Renal impairment: for those with pretransplant renal impairment, drug regimens with reduced CNI exposure maybe appropriate. (8) Comorbid diseases: diabetes mellitus or osteoporosis is exacerbated by steroids. Azathioprine is used in patients with inflammatory bowel disease.

Historically, CNI with an antimetabolite or mTORi became common practice to reduce renal impairment. The same CNI brand used in each recipient but few recipients exposed to diverse brands. Moreover, Prograf switched to Advagraf in cases that needed long-term high doses.

Target trough whole blood levels for the stable recipient are usually between 3 and 8 ng/L for tacrolimus and between 80 and 100 μg/L for cyclosporin. For trough CNI drug levels, the patients do not take their drug on the morning of the blood test. Potential drug-drug interactions are always considered. In general, serum levels of CNIs are kept at the upper therapeutic window within the first year unless side effects limit the goal and mTORi’s or MMF is added. After that, this goal is reduced to a mid-therapeutic window, with or without mTORi’s or MMF. The same goal levels are also used for mTORi’s.

### Study design and patients

The current research is a single-center retrospective study performed at the Liver Institute of the HMO, the largest hospital in the Jerusalem district.

### SARS-CoV-2 positive group

All SARS-CoV-2 positive LT recipients were included in this group. Criteria for hospitalization followed the same indications as nontransplant cases. In the first pandemic year (until November 2020), the regimen of immune suppression in 13 hospitalized severe patients switched to Dexamethasone monotherapy at an initial dose of 6 mg a day for 5 to 7 days or until stabilization; then, the modified baseline regimen was re-administered. The sepsis protocol in HMO was stopped when newer literature reported that switching or decreasing immunosuppression led to increased mortality.^15^ Antiviral medication such as Remdesevir was used at the attending physician’s decision. Asymptomatic SARS-CoV-2 positive cases were identified during the serology screen in the prevaccination assessment.

### The vaccine group

This group included liver recipients at the HMO Liver Institute who underwent documented vaccination and had serologic reevaluation at least 1 week after the vaccine was completed.

Vaccine response was defined as serum anti-S levels of ≥19 AU/mL without postvaccine SARS-CoV-2 infection. Vaccine failure was defined as serum anti-S levels of <19 AU/mL or postvaccine SARS-CoV-2 infection.

### Patient’s data

Patient data were extracted and collected from the electronic HMO medical system. Data included patient age, gender, body mass index, primary liver disease before transplant, time, and events from transplant to SARS-CoV-2 (infection or vaccination). The immunosuppressive regimen included and the dosages were unified, different steroids were unified by prednisone and MMF by Cellcept. Moreover, we included serum levels, metabolic background and treatment, complete blood count with international normalized ratio, partial thromboplastin time, and comprehensive biochemistry. In SARS-CoV-2 positive patients, clinical SARS-CoV-2 manifestations, management of immunosuppressant agents, antiviral therapy during hospitalization, and patient outcomes were collected. The anti-S IgG was not assessed in nonvaccine nor vaccine groups after recovery.

The study protocol conformed to the ethical guidelines of the 1975 Declaration of Helsinki and was approved by the local HMO Ethics Committee (Trial registration number: 1000-20-HMO). The local ethical committee waived informed consent since the vaccine was standard of care practice. All authors had access to the study coded data (without personal details), and reviewed and approved the final manuscript.

### Statistical analysis

For descriptive analysis, we used counts and percentages for categorical variables. Continuous variables are summarized as mean and SD. *t* test was used to compare means of continuous variables. Mann-Whitney and χ^2^ were used to compare between groups as appropriate. Univariate and multivariate logistic regression models were utilized to evaluate the impact of vaccination by test variables (clinical, laboratory, and immunosuppression). A *p* value of <0.05 was considered statistically significant. All statistical analyses were performed using the SPSS statistical package Version 23.

## RESULTS

### Patient characteristics

From March 1, 2020 to September 25, 2021, we included 167 cases. This was the total study population. The study excluded patients with low or difficult compliance and lacked anti-S serology or other essential medical information.

The mean age of the total study population was 57±14.8 years, post-transplant follow-up was 10.1±7.8 years, 61.7% were males, 66.5% were Jewish, mean body mass index was 27.2±5.9, and 4 patients died (2.4%) at the end of follow-up (3 due to SARS-CoV-2 complications and 1 due to post-transplant complications). Viral hepatitis B and C were the underlying etiology in 33.5%, fatty liver in 24%, autoimmune and cholestatic diseases (primary sclerosing and biliary cholangitis, drug-induced liver injury, and cholestatic genetic diseases) in 28.1%, metabolic disorders (Wilson’s disease and other congenital etiologies) in 6% and cryptogenic disease in 6.6% of the cases. The direct indication for transplantation was decompensated cirrhosis in 94%, HCC in 11.4%, fulminant hepatic failure in 3.6%, and 1% metabolic disease for gene therapy (without cirrhosis or HCC).

#### The total SARS-CoV-2 positive recipients

**FIGURE 1:**
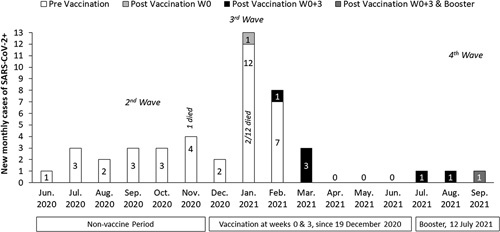
The monthly distribution of SARS-CoV-2 positive liver transplant (LT) recipients during the 4 SARS-CoV-2 waves in Israel. White bars represent 37 LT recipients who had SARS-CoV-2 infection before vaccination, including 21 who were infected during the early vaccine program period (December 2020-February 2021). Eight recipients experienced SARS-CoV-2 infection after vaccinations during the third and fourth pandemic waves. The first case in January 2021 (illustrated by the light gray bar) is a single case that received only the first vaccine 3 weeks before SARS-CoV-2 diagnosis. From February to September, 7 recipients had SARS-CoV-2 infection 10 to 55 days after vaccination, 6 cases after the first 2 vaccines (W0, and W3, black bars, 2 cases had low anti-S levels of 34.3 and 69.7 AU/mL, 4 cases were vaccine failure) and the eighth case 1 month after the booster in September (dark gray) was a serologic failure during the 3 doses. Three out of 45 LT cases who had SARS-CoV-2 infection died.

To assess the dynamic impact of vaccination on SARS-CoV-2 infection, 45/167 recipients (26.9%) with a SARS-CoV-2 infection were included (Fig. 1). Thirty-seven cases (22.2%) experienced SARS-CoV-2 infection before the vaccine program started in December 2020 and January 2021. While 34/37 patients had symptomatic active disease, 3 cases had serologic evidence for asymptomatic infection. The additional 8 cases (4.8%) had SARS-CoV-2 disease after vaccination. Of these 8 patients, the first 5 were confirmed in the third SARS-CoV-2 wave, 10 to 54 days after receiving the last vaccine dose (1 case after the first and 4 after 2 doses). As the vaccination program continued, from April to June 2021, there were no recorded SARS-CoV-2 infections among recipients, agreeing with the general outcome in Israel during this period. However, during the Delta variant fourth wave, additional 3 vaccinated liver recipients had SARS-CoV-2 infection almost 6 to 8 months after the last vaccine dose, a case per month from July to September 2021. A month after the booster vaccine, 1 of the 3 cases from the fourth wave was confirmed with SARS-CoV-2 infection; this was a serologic vaccine failure for all 3 doses. Two cases did not get the third vaccine launched in early July 2021. One was a serologic vaccine failure following the first 2 vaccines (anti-S titers of 3.9 AU/mL each), and the other had an inadequate serologic response of 69.7 AU/mL. Therefore, the total SARS-CoV-2 positive group of 45 LT recipients was subdivided into prevaccination (37 cases) and postvaccination (8) subgroups (Table 1).

**TABLE 1:** Patient characterization of the SARS-CoV-2 positive recipients (prevaccination vs. postvaccination) and the vaccinated recipients (failure vs. vaccine responders)

	SARS-CoV-2+			Vaccine response		
Number, (%)	Prevaccination 37/167 (22.2%)	Postvaccination 8/167 (4.8%)	*p*	Failures (38+8) 46/130 (35.4%)	Responders 84/130 (64.6%)	*p*
(A) Clinical patient characterization						
Characterization						
Age, years	52.3±18.3	59.5±10.7	NS	60.4±12	57.1±14	0.09
Sex, males (%)	45.90	87.50	0.017	65.20	66.70	NS
Jew (%)	48.60	62.50	NS	71.70	71.40	NS
BMI, kg/m^2^	28.5±7.4	27.1±5.6	NS	27.5±6.8	26.4±4.5	NS
Post-LT FU, years	10.6±7.2	8.1±10.2	NS	5.8±8.2	12.1±8.5	<0.0001
Death at end of FU (%)	8.10	0	NS	2.20	0	0.09
Smokers (%)	2.70	25.00	0.01	17.40	14.50	NS
Hypertension (%)	32.40	25.00	NS	33.30	34.90	NS
DM/IFG (%)	48.60	12.50	0.031	31.10	36.10	NS
Transplant etiology (%)						
Viral hepatitis B&C	27.00	37.50	NS	32.60	36.90	NS
NAFLD/AFLD	24.30	0	0.062	23.90	23.80	NS
AIH, PBC, PSC, others	21.60	37.50	NS	34.70	32.2	NS
Metabolic	16.20	12.50	NS	2.20	3.60	NS
Cryptogenic	10.80	12.50	NS	8.70	3.60	NS
HCC	5.40	12.50	NS	15.20	11.90	NS
FHF	5.40	0.00	NS	4.30	2.40	NS
(B) Laboratory patient characterization						
Hematology						
WBC, K/µL	5.8±2.6	7.5±3.5	0.060	6.4±2.7	6.5±2.3	NS
HGB, g/dl	11.8±2.4	11.9±2.1	NS	12.2±2	13.5±1.9	0.0002
PLT, ×10^9^/L	176.9±83.1	196.1±74.7	NS	185.9±111.3	180±74.7	NS
INR, ratio	1.2±0.6	1.0±0	NS	1.2±0.4	1.1±0.2	0.06
Biochemistry						
ALT, U/L	100.3±442	20.5±11.4	NS	23.6±13.8	35.2±46	0.048
AST, U/L	228.7±1183.6	21.0±5.5	NS	23.7±14.4	33±24	0.009
ALP, U/L	132.1±97.5	97.3±37	NS	126.8±70.6	130.7±82.6	NS
GGT, U/L	89.5±169.4	41.6±27	NS	78±111.4	70±149.7	NS
Total bilirubin, μmol/L	9.5±5.7	12±7	NS	13.5±15.2	14±11.6	NS
Albumin, g/L	39±6.8	41.4±5.6	NS	41.2±5.5	40±9.5	NS
FBG, mmol/L	7.4±4.2	5.3±1.1	NS	10.3±28	6.5±2.1	NS
HBA1c (%)	6.6±1.6	6.6±0.8	NS	6.8±1.4	6.2±1.6	NS
TG, mmol/L	1.7±1.1	2.5±1.6	0.049	2.1±1.5	2±2.8	NS
HDL, mmol/L	1.3±0.5	0.9±0.2	0.011	1.1±0.4	1.3±0.5	0.01
LDL, mmol/L	2.3±0.7	2.6±1.3	NS	2.5±1.1	2.5±1	NS
Anti-S Ab’s, AU/ml				8.2±10.5	185±185.5	<0.0001
Metabolic therapy						
Statins (%)	32.40	25.00	NS	21.70	26.50	NS
GLA (%)	45.90	12.50	0.042	34.80	26.50	NS
(C) The immunosuppression regimen of the subgroups						
Steroid (%)	43.20	37.50	NS	52.20	27.40	0.002
Dose, mg	7.9±9.6	10.0±5	NS	9.9±10.1	7.3.0±5.9	NS
mTOR inhibitors						
Everolimus or sirolimus (%)	43.20	12.50	0.054	17.40	29.80	0.062
Sirolimus (%)	8.10	0	NS	0	7.10	0.032
Dose, mg	1.7±1.2	0	NS	0	1.2±0.4	0.039
Levels, ng/mL	7.5±5.4	0	NS	0	7.7±3.6	0.061
Everolimus (%)	35.10	12.50	NS	17.40	25.00	NS
Dose, mg	1.8±0.9	0.8±	NS	1.5±0.7	1.4±0.6	NS
Levels, ng/mL	5.0±4.2	2.2±0	NS	3.2±1.7	2.5±1	NS
MMF (%)	27.00	62.50	0.028	69.60	25.60	<0.0001
Dose, mg	1050.0±685.2	1600.0±418.3	NS	1484.4±515.6	1046.2±504.8	0.002
Calcineurin inhibitors						
Cyclosporine or FK506 (%)	86.5	87.5	NS	95.7	84.5	0.029
Cyclosporine (%)	2.70	0	NS	6.50	8.30	NS
Dose, mg	25±0	0	NS	91.7±62.9	105.6±79.5	NS
Levels, ng/mL	49.0±0	0	NS	77.8±10.2	66.5±20.4	NS
FK506 (%)	83.80	87.50	NS	89.10	76.20	0.037
Dose, mg	2.7±1.7	2.7±1.8	NS	3.3±2.4	2.1±1.6	0.001
Levels, ng/mL	5.6±2	5.4±2.7	NS	6.5±3.2	5.2±2.9	0.019
UDCA (%)	37.80	37.50	NS	34.80	32.10	NS

*Notes:* This table showing clinical (A), laboratory (B), and therapeutic (C) characterizations of SARS-CoV-2 positive recipients and vaccinated recipients. Values are presented as average±SD or rates as percentages.

(A) Age and postliver transplantation follow-up (FU) are shown as years. Body mass index (BMI) in kg/m^2^. Rates for death at the end of follow-up, male gender and Jewish ethnic background, hypertension, diabetes mellitus (DM), and impaired fasting glucose (IFG) are presented as percentages. Etiologies for liver transplantation included viral hepatitis B and C (viral hepatitis B and C), NAFLD, autoimmune hepatitis (AIH), and cholestatic etiologies (primary sclerosing and biliary cholangitis, drug-induced liver injury, and cholestatic genetic diseases), metabolic etiologies (Wilson and genetic diseases), cryptogenic, HCC and fulminant hepatic failure (FHF) are presented as percentages.

(B) Laboratory results of the SARS-CoV-2 positive recipients are shown as a total group, prevaccination and postvaccination subgroups. Selected laboratory results include white blood cells (WBC), hemoglobin levels (HGB), platelet counts (PLT), international normalized ratio (INR), serum albumin levels g/L, INR, gamma-glutamyl transferase (GGT) U/L, alanine and aspartate aminotransferases (ALT, AST), alkaline phosphatase (ALP), GGT), total bilirubin, albumin, fasting blood glucose (FBG), glycosylated hemoglobin (HBA1c), triglycerides (TG) and HDL, LDL. Metabolic management includes statins and glucose-lowering agents (GLA).

(C) The medications of the immunosuppression regimen in the study group included steroids, mammalian target of rapamycin inhibitors (mTORi) including sirolimus or everolimus, mycophenolate mofetil (MMF), cyclosporine-A, FK506, and ursodeoxycholic acid (UDCA). Data are presented as the percentage of recipients treated with each medication, mean daily dose, and levels (±SD).

The mean age of the 45 SARS-CoV-2 positive recipients was 53.6±17.3 years (45.9% males, 51.1% Jews), mean body mass index was 28.2±7 kg/m^2^, post-transplant follow-up was 10.1±7.8 years. 6.7% were smokers, 31.1% had hypertension, and 42.2% had impaired glucose homeostasis. Most of these parameters, and the etiology for a LT, did not show significant changes between the prevaccination and postvaccination subgroups. However, the postvaccination subgroup were mainly males, and lacked an NAFLD background, with significantly lower rates of impaired glucose homeostasis (12.5% vs. 48.6%, *p*=0.03) but with higher smoking rates. The total death rate was 3/45 (6.7%), all from the prevaccination subgroup (3/37, 8.1%) but all the 8 vaccinated recipients fully recovered from SARS-CoV-2 infection (Table 1A).

The nonvaccinated subgroup, compared to the postvaccinated subgroup, had a significantly lower level of triglycerides (1.7±1.1 vs. 2.5±1.6 mmol/L, *p*=0.049, respectively) and high-density lipoprotein (1.3±0.5 vs. 0.9±0.2 mmol/L, *p*=0.01, respectively). Despite higher rates of impaired glucose homeostasis and the need for glucose-lowering agents in the nonvaccinated subgroup (GLA in 45.9% vs. 12.5%, *p*=0.04), glucose control was similar according to HBA1c levels (Table 1B).

Regarding the immunosuppression regimen (Table 1C), the prevaccination subgroup had significantly lower rates of MMF treatment (MMF in 27% vs. 62.5%, *p*=0.03) and a tendency to an increased need for mTOR inhibitors (either sirolimus or everolimus in 43.2% vs. 12.5%, *p*=0.054). These results support published data showing that lower rates of MMF predict worse outcomes.^15^

#### The total vaccinated group

The total vaccinated group included 130 prevaccination negative serum anti-S cases who received the SARS-CoV-2 vaccine. Of them, 129 completed both doses, and 1 patient received only the first vaccine dose, as he got infected with SARS-CoV-2 after 3 weeks, just before the second dose. All 130 cases were evaluated for vaccine efficacy by clinical follow-up and by assessment of serum anti-S at least 1 week following the second dose. This group was defined as the total vaccinated group. The 8/130 (6.2%) patients with a postvaccine SARS-CoV-2 infection described above were defined as clinical vaccine failure and overlap with the SARS-CoV-2 positive group (Fig. 1). Thirty-eight cases (29.2%) were serological vaccine failure, keeping the total vaccine failure rate 46/130 (35.4%). The remaining 84 (64.6%) cases were serological vaccine responders.

According to Israeli policy, a third vaccine booster was strongly recommended starting in July 2021. Data from patients receiving this booster was collected from 38 recipients (16 were vaccine failures).

#### Risk factors for vaccine failure

A multivariate analysis assessed the differences according to vaccine outcome (Table 1A–C). Vaccine failure (n=46) and vaccine responders subgroups (n=84) had similar rates of gender (males in 65.2% vs. 66.7%), origin (Jewish 71.7% vs. 71.4%), mean body mass index (27.5±6.8 vs. 26.4±4.5 kg/m^2^), rates of hypertension (33.3% vs. 34.9%), and impaired glucose homeostasis (31.1% vs. 36.1%). There was no significant difference in the transplantation etiologies (Table 1A). Only 1/130 of the vaccinated group died from post-transplantation complications, although it was a vaccine failure. The mean age and post-transplant follow-up period differed in both vaccine outcome subgroups. Vaccine failures tend to be older than responders (60.4±12 vs. 57.1±14 years, *p*=0.09). Interestingly, the postliver transplantation follow-up was significantly shorter in the vaccine failures than responders (5.8±8.2 vs. 12.1±8.5 years, *p*<0.0001).

Laboratory tests (Table 1B) showed that the vaccine failure subgroup had significantly lower hemoglobin (12.2±2 vs. 13.5±1.9 g/dL, *p*=0.0002), alanine transaminase (23.6±13.8 vs. 35.2±46 U/L, *p*=0.048), aspartate transaminase (23.7±14.4 vs. 33±24 U/L, *p*=0.009), and high-density lipoprotein (1.1±0.4 vs. 1.3±0.5, *p*=0.01). Other laboratory tests, and the use of statins and glucose-lowering agents, were similar in both groups.

**FIGURE 2:**
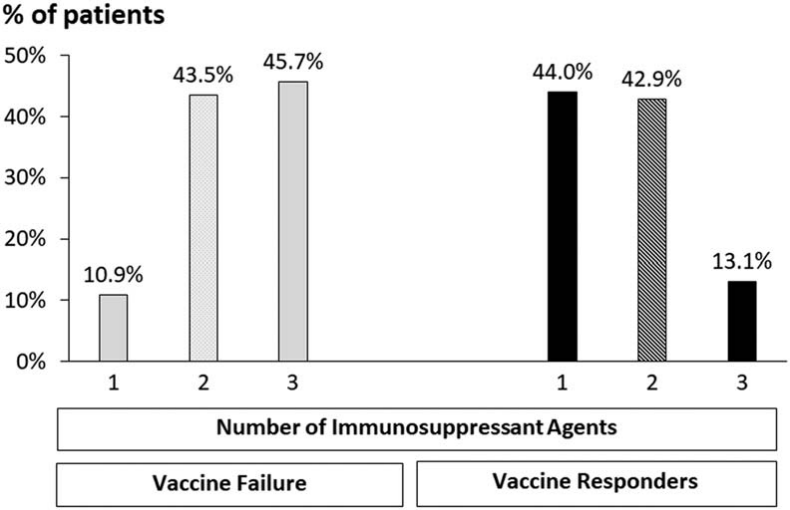
Number of drugs in immunosuppressant regimens given to the vaccinated subgroups. Bars represent the rate of recipients in each immunosuppressant regimen according to the number of drugs in the vaccine failures (gray bars) versus vaccine responders (black bars) subgroups.

The immune suppression regimen played an important role in the vaccine response (Table 1C). Larger rates of patients in the vaccine failure subgroup received steroids (52.2% vs. 27.4% responders, *p*=0.002), MMF (69.6% vs. 25.6%, *p*<0.0001) and CNIs (95.7% vs. 84.5%, *p*=0.029), mainly FK506 (89.1% vs. 76.2%, *p*=0.037). They also received higher dosages of MMF (1484.4±515.6 vs. 1046.2±504.8 mg, *p*=0.002) and FK506 (3.3±2.4 vs. 2.1±1.6 mg, *p*=0.001) and had higher serum levels of FK506 (6.5±3.2 vs. 5.2±2.9, *p*=0.019). On the other hand, the vaccine failure subgroup received fewer mTOR inhibitors (17.4% vs. 29.8%, *p*=0.062), mainly sirolimus (none vs. 7.1%). Furthermore, significantly larger rates of patients in the vaccine failure subgroup received multiple immunosuppressive agents (Fig. 2) than in the vaccine response subgroup who were predominantly treated with mono or double immunosuppressants (*p*<0.0001). A double or triple-drug regimen was given to 89.2% versus 56% of patients, while a single drug regimen was given to 10.9% versus 44%, respectively.

MMF and mTOR inhibitors are routinely used to treat more complicated cases than those treated with a low-dose monotherapy of CNIs. Thus, to better explore the role of mTOR inhibitors, we focused on a subanalysis of 81 LT recipients treated by either mTOR inhibitors (33 cases, 40.1%) or MMF basis (53, 65.4%), and 5 LT patients received both (Table 2). Similar to the general LT population (Table 1C), the subpopulation treated with either MMF or mTOR inhibitors also showed an inadequate serologic response that correlated with higher steroids, MMF, and CNIs (Table 1D). However, the use of mTOR inhibitors significantly correlated with improved vaccine response. mTOR inhibitors contributed to a good serologic response either directly or by reducing the FK506 dosages and serum levels in the subgroup of vaccine responders.

**TABLE 2:** The immunosuppression regimen based on MMF or mTOR inhibitors

	Vaccine failure (38)	Vaccine responders (43)	*p*
Steroid (%)	55.3	23.3	0.001
Dose, mg	10.5±10.7	9.1.0±6.8	NS
mTOR inhibitors (%)			
Everolimus or sirolimus	21.1	58.1	0.0003
Sirolimus	0	14	0.008
Everolimus	**21.1**	**44.2**	0.01
MMF (%)	**84.2**	**48.8**	0.0003
Dose, mg	1484.4±515.6	1046.2±504.8	0.002
Calcineurin inhibitors			
Cyclosporine or FK506 (%)	94.7	69.8	0.002
Cyclosporine (%)	7.9	7	NS
Dose, mg	91.7±62.9	100±0	NS
Levels, ng/mL	77.8±10.2	53.2±7.9	0.01
FK506 (%)	**86.8**	**62.8**	0.007
Dose, mg	3.1±2	2.4±2.1	0.08
Levels, ng/mL	6.5±3.5	4.8±2.9	0.044
UDCA (%)	36.80	37.2	NS

*Notes:* The immunosuppression regimen based on MMF or mTOR inhibitors of the vaccinated recipients is shown as vaccine failure versus vaccine responders subgroups.

The immunosuppression regimen of the vaccinated recipients shown as total group, vaccine failure, and vaccine responders subgroups, shown as percentage, daily dose and levels. Regimens include: steroids, mammalian target of rapamycin inhibitors (mTORi) including sirolimus or everolimus, mycophenolate mofetil (MMF), cyclosporine-A, FK506, and urdodeoxycholic acid (UDCA).

#### Long-term durability of anti-S and responses to boost vaccine

Of the 38 cases of serologic vaccine failure after the first 2 vaccine doses, results of long-term anti-S assessment were available for 25 recipients retested before the third vaccine (according to the July 12 ministry approval for SOTR). Thus, anti-S was assessed shortly after the second dose (>7 d) and at a longer interval of 102.6±43.3 days between the second and third vaccines. Long-term follow-up showed that only in 1 recipient of the 25 failures, anti-S titers rose from 6.8 to 22.1 AU/mL; the other 24 cases remained serologic failure. However, following the booster vaccination of the failures, available results showed that 5/17 (29.4%) tested after the booster (third vaccine) increased anti-S titers to >200 AU/mL; the rest remained <12 AU/mL. One of these demonstrated serologic failure following the 3 vaccine doses had SARS-CoV-2 infection in September 2021 (Fig. 1, dark gray bar), occurring 1 month after the booster. SARS-CoV-2 infection in this case was 3.3 years after transplantation due to primary sclerosing cholangitis, with an excellent post-transplant course, treated with daily doses of 1500 mg MMF and 1.5 mg FK506 keeping low FK506 blood levels of 4 ng/mL.

Out of the 84 cases with a protective serologic vaccine response achieving anti-S ≥19 AU/mL at least 1 week after second vaccine dose, long-term anti-S results were available for 27 recipients who were retested 110.4±43.6 days after the second vaccine, just before the boost vaccine dose. At longer follow-up, 5/27 (18.5%) were found to have developed a serological failure. Short-term anti-S <100 AU/mL was the risk factor for losing serologic response in the longer term. However, the remaining 22 kept in the vaccine response range. Of the 22 recipients tested before and after the boost dose, 1 case was vaccine failure before the boost and remained so after the boost. Out of the 21 responders, 5 recipients had pre-boost anti-S titers ≥200 AU/mL, and 16 had titers <200 AU/mL. After the booster, 19 increased anti-S titers to ≥400 AU/mL and 2 to 347 AU/mL.

## Discussion

Because the currently authorized vaccine focuses on response to the spike protein, an immune response to the virus might be detected after natural infection and successful vaccination.^16^ The immunosuppression SOTR inhibit the response by various mechanisms of T-cell and B-cell function. Therefore, immunosuppression affected the severity of SARS-CoV-2 infection and the efficacy of vaccination in LT recipients.

The SARS-CoV-2 positive group in the current study included 45 LT recipients from June 2020 to September 2021 (Fig. 1), with an infection peak of 26 LT cases (57.8%) from December 2020 to March 2021. These 4 months were also the epidemiologic peak in Israel. The 26 cases comprised 21 prevaccinated and 5 postvaccinated SARS-CoV-2 positive LT recipients. The 21 prevaccinated cases were 56.8% of the 37 nonvaccinated SARS-CoV-2 LT recipients that became positive during the early vaccine period from December 2020 to February 2021. Those 21 patients hesitated and did not get vaccinated early, despite vaccine availability. However, following the progress of the vaccine program in Israel and flattening the curve data of SARS-CoV-2 to almost 0, no recorded SARS-CoV-2 in LT was observed for 3 months, from April to June 2021. That was the direct result of vaccination in the general population, including the LT recipients. SARS-CoV-2 disappearance occurred despite the background of low immunogenicity and a serologic response rate of only 65% in LT recipients (Table 1A).

Although April to June 2021 was a SARS-CoV-2 epidemiologic peak in Israel (recognized fourth wave with the Delta variant), only 3 new LT cases developed a mild SARS-CoV-2 infection. Collectively, we had 8 postvaccinated SARS-CoV-2 positive LT recipients. Therefore, SARS-CoV-2 declined from 22.2% to 4.8% after vaccine administration.

Three of the 37 nonvaccinated SARS-CoV-2 positive LT recipients died. One death was from the 16/37 cases with SARS-CoV-2 infection before the vaccine was available (1/16). The other 2 deaths of patients were from the 21/37 cases who experienced SARS-CoV-2 infection shortly after vaccine availability (2/21) and were potentially preventable. None of the 8 postvaccinated SARS-CoV-2 positive LT recipients died. The low numbers and the mild disease severity in the postvaccinated SARS-CoV-2 positive LT recipients reflect a positive impact of the vaccine. Despite less favorable lipid control and smoking rates, the good outcome in the postvaccination SARS-CoV-2 positive subgroup (Table 1A, B) is probably a direct result of the vaccination.

The impaired immunogenicity could result from vaccine failure following the first 2 doses, the loss of vaccine response among those with low immunogenicity (anti-S <100 AU/mL), or the rapidly spreading variants of SARS-CoV-2.^17^ Although the 3 LT patients recorded from April to June 2021 had low or no immunogenicity, we also observed a long-term loss of vaccine response in our general population. A pilot of 27 LT cases with a positive serologic vaccine response after the second vaccine dose was reassessed after a mean follow-up of 110.4±43.6 days before the booster dose. Five patients (18.5%) lost response, and 4 became borderline (14.8%). All 9 cases had a short-term response of <100 AU/mL, but the remaining 25 patients were in the vaccine response range. The data suggest that the response’s durability depends on the initial response following the second vaccine dose. On the other hand, 24/25 LT recipients with serologic vaccine failure after the first 2 vaccine doses maintained a serologic failure when retested after 102.6±43.3 days (only 1/25 LT recipients rose to borderline anti-S titers).

We suggest that the booster vaccination of the population in Israel, including our LT recipients, contributed to lowering the SARS-CoV-2 burden during the fourth wave. Our booster pilot with the third vaccine showed a good response. Of the 22 LT responders after the second dose, tested before and after the boost, 21 responders benefited from the booster, and only 1 case was a vaccine failure and remained so after the boost. The data suggests a robust response following the booster among the early responders after the second dose. However, 5/17 (29.4%) with vaccine failure increased anti-S titers >200 AU/mL following boost vaccination, and the rest remained <12 AU/mL. Our results are in line with reported experience.^18^

The current study supports the reported finding that the immune response elicited by the Pfizer’s BNT162b2 vaccine is affected by immune suppression. Impairment of the immune response in LT recipients is associated with increased numbers, dosages, and serum levels of steroids, CNIs, and MMF (Table 1C, D, Fig. 2). However, mTOR inhibitors tend to achieve a favorable vaccine response (Table 1c). Therefore, we focused on recipients treated with either mTOR or MMF, as both are used as an addition or to spare CNIs. Unlike the lower response with MMF, mTOR inhibitors were significantly associated with a good vaccine response (Table 2D). These findings agree with reports that the SARS-CoV-2 spike promotes inflammation and apoptosis through autophagy by ROS-suppressed PI3K/AKT/mTOR signaling.^19^ Moreover, immunoregulation with mTOR inhibitors was also reported to prevent SARS-CoV-2 severity.^20^

Transplant societies recommend maintaining stable immunosuppressive regimens, including antiproliferative agents (such as MMF) in patients receiving SARS-CoV-2 vaccination.^9^ Alteration of immunosuppression outside the clinical trial setting is discouraged because of the potential risk of rejection. However, the current study and others show low vaccine responses in mycophenolate-treated patients and unaffected or improved responses in everolimus-treated patients. Thus, we suggest switching from mycophenolate to everolimus before the next vaccination in selected situations. In particular, before the booster in cases of vaccine failure and when no risk from switching is expected. Switching MMF to everolimus might improve immunogenicity, but further studies are needed to detect the impact of this change.

In conclusion, besides the reported impaired immunogenicity of the Pfizer’s BNT162b2 vaccine, the current study shows a clinical failure in 8 LT recipients. The vaccination program alleviated SARS-CoV-2 severity among LT recipients. Improving the vaccine response is a goal for the safety of LT recipients during the SARS-CoV-2 pandemic. Therefore, we defined risk factors for a failed response. The current study also shared basic risk factors with reported data—the longer post-transplantation follow-up associated with SARS-CoV-2 vaccine response and less consumption of immunosuppressants. Steroids, FK506, and MMF were predictors of vaccine failure. As expected, the stronger immunosuppression was significantly associated with lower transaminases. On the other hand, mTOR inhibitors may improve vaccine response. Finally, booster vaccines are a suggestive strategy to maintain a good response. Although the number of LT recipients tested after the booster was low, the boost dose significantly increased titers and may secure a better and maybe longer protection.

This study presents proof that a booster vaccine 5 months after the second dose increased immunogenicity in both failure and responder subgroups of LT recipients, in addition to increasing the chances of blocking infection.
